# Bilateral Bilothorax: An Unusual Cause of Bilateral Exudative Pleural Effusion

**DOI:** 10.7759/cureus.5185

**Published:** 2019-07-21

**Authors:** Kanval Shah, Nakul Ravikumar, Q. Kamran Uddin, William McGee, Mary Jo S Farmer

**Affiliations:** 1 Internal Medicine, University of Massachusetts Medical School - Baystate Medical Center, Springfield, USA; 2 Pulmonary and Critical Care, University of Massachusetts Medical School - Baystate Medical Center, Springfield, USA; 3 Critical Care, University of Massachusetts Medical School - Baystate Medical Center, Springfield, USA

**Keywords:** bilateral bilothorax, pleural effusion

## Abstract

Bilothorax is an uncommon cause of exudative pleural effusion; the majority of reported cases are right-sided while a bilateral presentation is extremely rare. The majority of cases are secondary to biliary obstruction, an extension of hepatic infections, and iatrogenic complications following percutaneous procedures or surgical interventions. Imaging studies and a diagnostic pleural tap can confirm the diagnosis. Early recognition and complete drainage are important to prevent life-threatening complications, including empyema formation. We present a case of a 71-year-old female who developed bilateral bilothorax as a complication of gallstone pancreatitis.

## Introduction

Bilothorax is the presence of bile in the pleural space. The first reported case of bilothorax was a young man who developed right-sided bilothorax following blunt trauma in 1971 [1]. Most cases of bilothorax are located on the right side. We could find only one other reported case of bilateral bilothorax, which was associated with biliary peritonitis [2]. Our patient initially presented with acute cholecystitis and gallstone pancreatitis, rapidly developed respiratory failure, and was subsequently diagnosed with bilateral bilothorax.

## Case presentation

A 71-year-old obese female presented to the hospital with acute onset abdominal pain accompanied by chills and fevers for 24 hours. The pain was sharp and shooting; it started in the right upper abdomen and spread to both shoulder joints. The pain was associated with nausea and one episode of bilious vomiting. The patient denied cough, chest pain, and urinary symptoms. She was a retired schoolteacher, a non-smoker, and denied excessive alcohol intake. There was no history of abdominal surgery.

The patient was afebrile with normal vital signs. Physical exam revealed clear lungs to auscultation, significant right upper quadrant tenderness, guarding, and positive Murphy’s sign to palpation. Lab work was remarkable for an elevated lipase and bilirubin (Table 1).

**Table 1:** Laboratory values WBC: white blood cell; AST: aspartate aminotransferase; ALT: alanine transaminase

Laboratory values (reference range)	Day of admission	Repeat labs( day 3)
WBC count (4.0- 11.0 k/mm^3 ^)	11.6 k/mm^3^	9.1 k /mm^3^
Alkaline phosphatase (35-104 units/L)	224 units/L	95 units/L
Lipase (13-60 units/L)	4510 units/L	114 units/L
AST (0-32 units/L)	122 units/L	21 units/L
ALT (0-33 units/L)	227 units/L	43 units/L
Total and direct bilirubin (0-1.2 mg/dl and 0-0.3 mg/dl)	5.9 and 4.1 mg/dl	1.4 and 1.0 mg/dl

**Figure 1:**
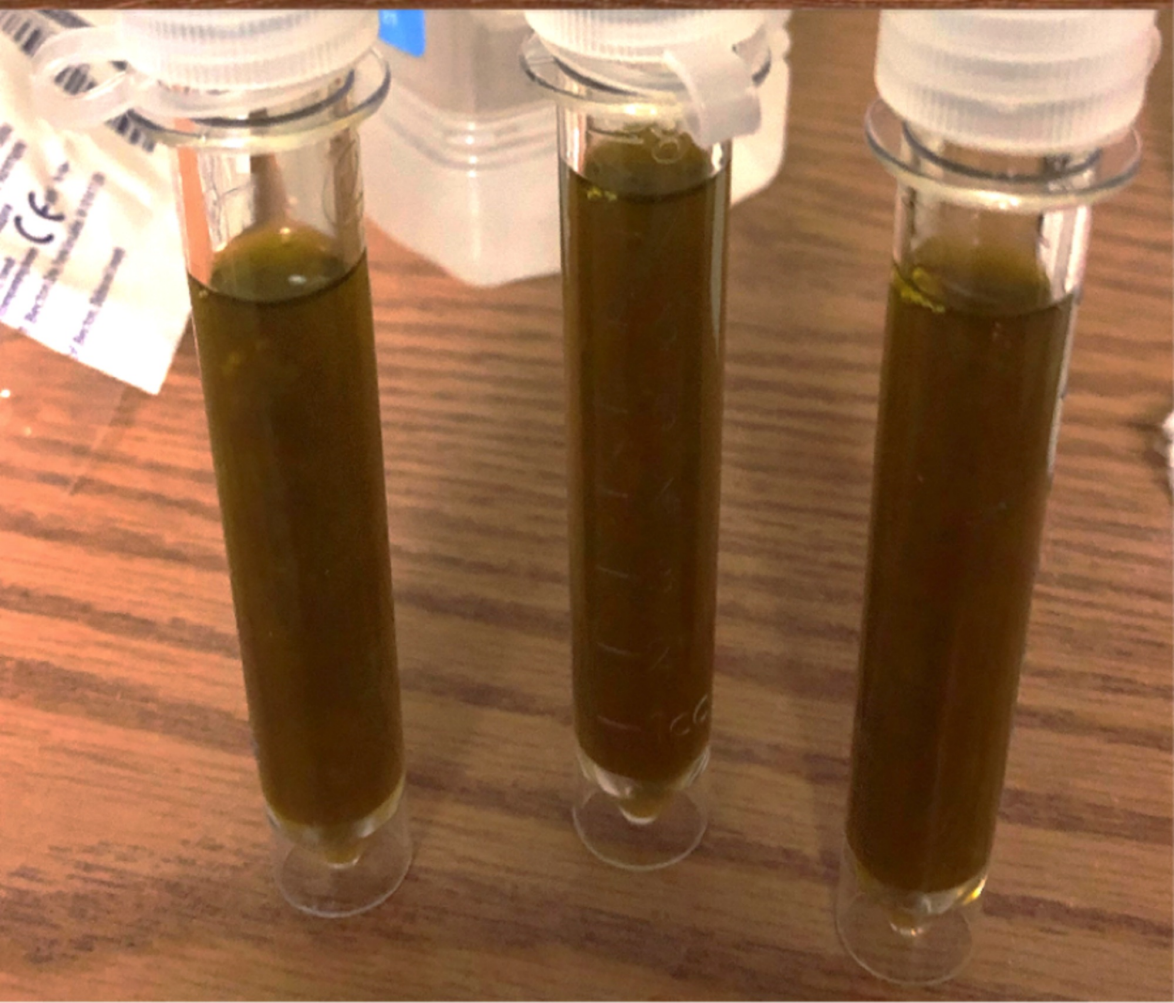
Left-sided pleural fluid samples showing bile pigments

A right upper quadrant ultrasound (US) showed multiple gallstones and gallbladder wall thickening of 8 mm, consistent with acute cholecystitis. The common bile duct was dilated at 0.8 cm. Intravenous fluids and piperacillin-tazobactam were initiated. Interventional radiology-guided percutaneous cholecystostomy tube placement was performed and repeat US showed successful decompression of the previously distended gall bladder. Liver function tests and lipase levels rapidly improved (Table 1). The patient subsequently developed acute respiratory distress and was intubated for hypoxemic respiratory failure. A computed tomography (CT) scan of the chest without contrast showed bilateral moderate to large pleural effusions. A left-sided thoracentesis was performed with drainage of 300 ml of cloudy greenish appearing fluid (Figure 1).

**Figure 2:**
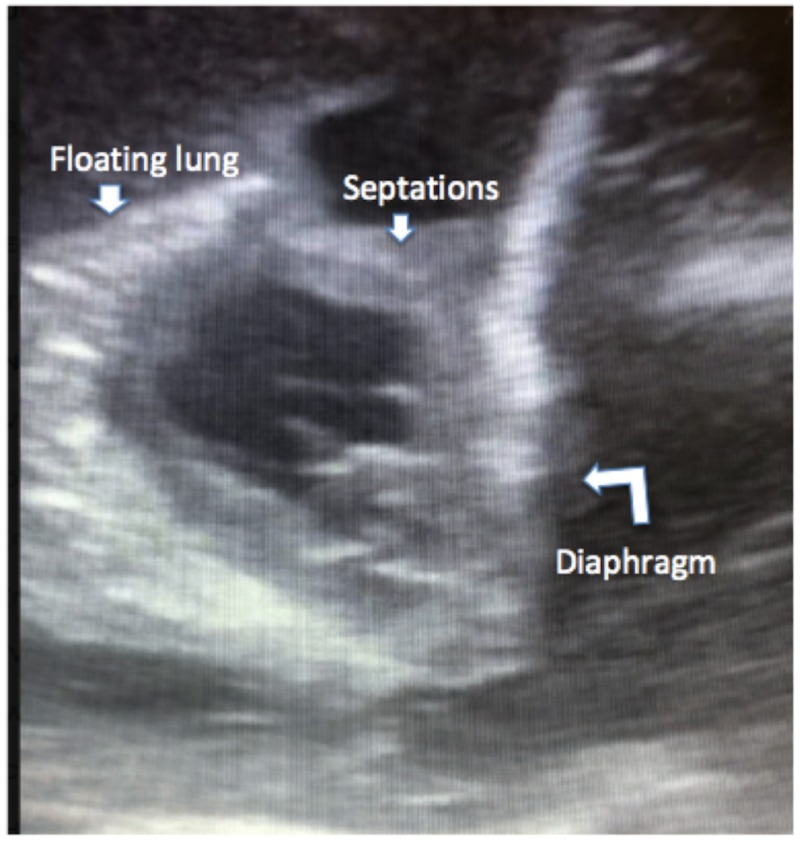
Ultrasound of the right lung Bedside ultrasound of the right lung showing complex fluid collection with multiple septations and lung ‘floating’ in pleural fluid

Bedside ultrasound of the right chest showed complex fluid collection with septations (Figure 2).

A right-sided chest tube was inserted with immediate drainage of 1700 ml of cloudy, green-colored fluid. A left-sided chest tube was placed with further drainage of 450 ml of similar appearing fluid. The pleural fluid analysis was consistent with an exudative etiology as per Light’s criteria (Table 2).

**Table 2:** Pleural fluid analysis WBC: white blood cell

Pleural fluid	Right side	Left side
WBC count (per cc^3^)	288	771
pH	7.62	7.37
Pleural fluid / serum protein (gm/dl)	2.4 / 4.8	2.9 / 4.8
Glucose (mg/dl)	57	98
Pleural fluid / serum LDH (units/ L)	2,781 / 376	2,810 / 376
Bilirubin (mg/dl)	4.7	9.1

**Figure 3:**
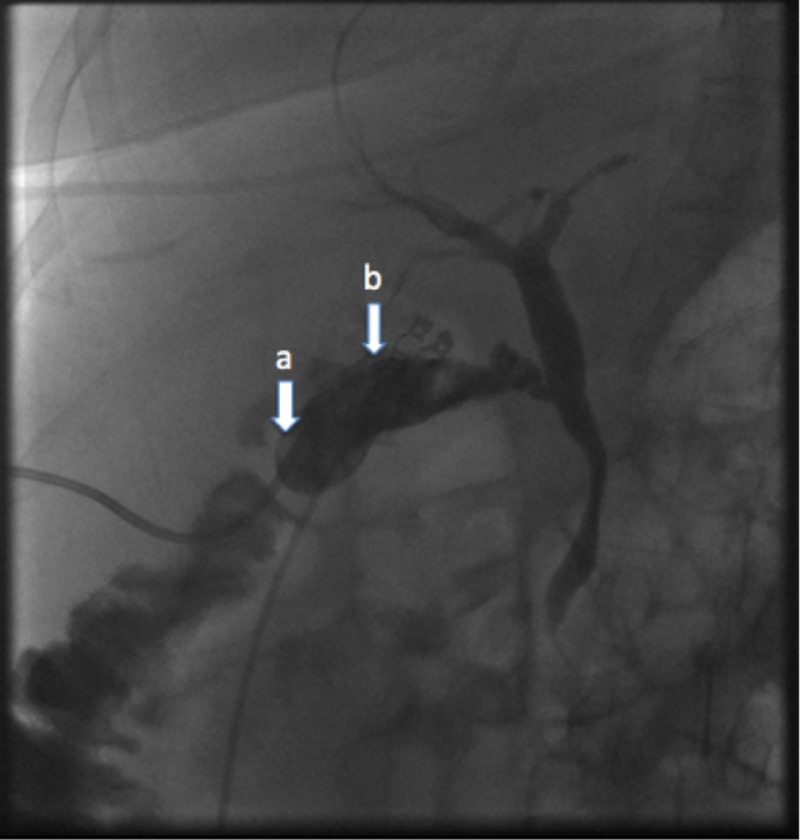
Cholecystogram Cholecystogram showing the indwelling cholecystostomy catheter (a) in a satisfactory position within the contracted gallbladder lumen (b) and prompt egress of contrast into the duodenum with no evidence of a bile leak

Pleural fluid/serum total bilirubin ratio was 3.9 on the right side (4.7: 1.2 mg/dl) and 7.5 on the left side (9.1:1.2 mg/dl). The microbiology culture was negative. As the total pleural fluid and serum bilirubin ratio was noted to be greater than 1.0 on both sides, a diagnosis of bilateral bilothorax was established. Given the presentation with acute cholecystitis and recent percutaneous procedure, the differential diagnosis included the extension of a subphrenic abscess, gastric perforation, and iatrogenic fistula formation. Further investigation, including computed tomography (CT) abdomen, upper gastrointestinal (GI) series, hepatobiliary iminodiacetic (HIDA) scan, and cholecystogram (Figure 3) were performed and were unrevealing.

**Figure 4:**
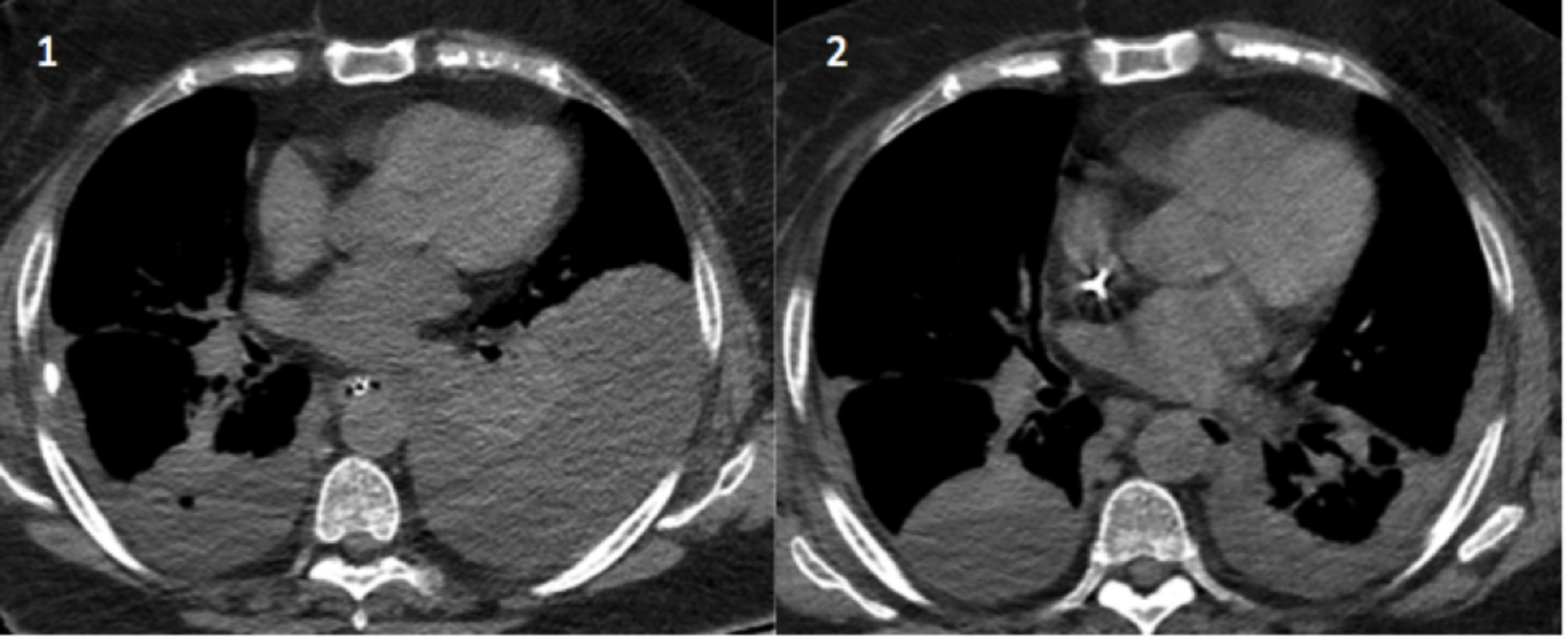
Chest CT scans Before (1) and after (2) chest computed tomography (CT) images following the administration of alteplase through the left chest tube into the pleural space

On Day 3, decreased output was noted from the left-sided chest tube with persistent radiographic findings of a moderate-sized pleural effusion. Ten milligrams of alteplase with 40 ml of normal saline was irrigated into the pleural space through the left chest tube with immediate drainage of 1200 cc of pleural fluid (Figure 4).

Bilateral chest tubes continued to drain well and were removed on Day 6 with complete resolution of pleural effusions on chest X-ray. The patient was successfully extubated and discharged to home with an indwelling cholecystostomy tube.

She underwent laparoscopic cholecystectomy four months after admission to the hospital. Our patient tolerated the procedure well and walked out of the hospital on recovery.

## Discussion

There are several possibilities for how bile travels into the pleural space, which includes the passive movement of bile through the diaphragm or lymphatic channels, traumatic or congenital defects in the diaphragm, and bilious fistulas. Other possible etiologies include the extension of biliary peritonitis, blunt trauma causing a biliopleural fistula, or a complication of open or percutaneous hepatobiliary procedures [3-6].

The diagnosis of pleural effusion can be made with a careful physical exam and conventional imaging studies, including chest X-ray, US, and CT scan. The diagnosis of bilothorax requires a high index of clinical suspicion and pleural fluid analysis. The most specific diagnostic feature is a fluid-to-serum bilirubin ratio greater than 1.0 [7]. Once the diagnosis of bilothorax is established, the next step is identifying the portal of entry of bile into the pleural space. Multiple diagnostic modalities have been described to evaluate the cause of bilothorax, including a HIDA scan [8]. Less commonly, laparotomy has been performed as a diagnostic and potentially therapeutic option in cases of surgically repairable fistulas [9].

In our case, given the patient’s initial presentation with acute cholecystitis, a CT scan of the abdomen was performed to rule out a subphrenic abscess. Cholecystogram was performed to evaluate for possible bilious fistula formation. We believe the bilateral bilothorax was a result of passive movement of bile through the diaphragm or lymphatic channels into the pleural space in the setting of obstructive jaundice, as no anatomic reason was found.

We noted decreased drainage on the left side from the chest tube and hence repeat imaging was performed, which confirmed the chest tube in the correct position. Alteplase, along with normal saline, was administered through the chest tube and clamped for two hours. Zuckerman et al. describe the use of tissue plasminogen activator for the management of complex pleural effusions without the risk of hemorrhagic complications [10].

There are no set guidelines on the management of bilothorax. Typical management includes immediate and complete drainage, as bilothorax has a high propensity to be associated with empyema.

## Conclusions

Bilateral bilothorax is a rare diagnosis, which requires a high index of clinical suspicion. Prompt diagnosis, correction of underlying cause, and complete drainage are all important for successful treatment.
